# Cleaning and Conditioning of Contaminated Core Build-Up Material before Adhesive Bonding

**DOI:** 10.3390/ma13122880

**Published:** 2020-06-26

**Authors:** Karsten Klosa, Walid Shahid, Milda Aleknonytė-Resch, Matthias Kern

**Affiliations:** 1 Department of Prosthodontics, Propaedeutics and Dental Materials, School of Dentistry, Christian-Albrechts University, 24105 Kiel, Germany; walidshahid@gmx.de (W.S.); mkern@proth.uni-kiel.de (M.K.); 2 Institute of Medical Informatics and Statistics, University Hospital Schleswig-Holstein, Campus Kiel, 24105 Kiel, Germany; resch@medinfo.uni-kiel.de

**Keywords:** core build-up material, saliva, silicone, cleaning, contamination, conditioning

## Abstract

The objective of this study was to evaluate the effects of different cleaning and conditioning procedures after contamination on the tensile bond strength (TBS) of a luting resin to a core build-up composite resin. Specimens (*n* = 384) made of a core build-up material were stored for 3 weeks in 37 °C water. Half of the specimens were contaminated with saliva and a disclosing silicone and then cleaned either using phosphoric acid, a pumice suspension, air-abrasion with alumina or polishing powder. Surface conditioning was performed by either using a dentin adhesive, a silane containing primer or a composite resin primer, which resulted in 24 unique combinations of 16 specimens per group. Before measuring TBS, half of the specimens of each group were stored in 37 °C water for 3d or were artificially aged for 150 days. Results show that cleaning with pumice or air-abrasion are superior methods compared to using a polishing powder or phosphoric acid. Silane is an inferior conditioning agent compared to composite or dentin primers. Ideally, after contamination, bonding surfaces should be cleaned with a pumice suspension and conditioned with a dentin adhesive. Those surfaces could also be cleaned and conditioned with air-abrasion with alumina particles and a composite resin primer.

## 1. Introduction

Fabrication of indirect dental restorations requires specific procedures including preparation of the tooth following explicit rules [1,2,3]. Due to the fact that cariogenic defects do not follow these rules, teeth often need to be filled using core build-up material prior to the preparation [4]. Whereas conventional cementation methods are relatively technically uncritical, the long-term success of an adhesive cementation depends on many factors such as material-specific conditioning and the adequate cleaning of the bonding surfaces after contamination [5,6,7]. These factors are well known and examined regarding tooth hard tissues and dental restoration materials such as alloys and ceramics, but little is known of the effects of these factors regarding core build-up materials [8,9,10,11].

During preparation and try-in procedures of dental restorations, bonding surfaces, i.e., tooth structures, restoration materials and core build-up materials might be contaminated by saliva [12,13], blood [14], dentin liquor and/or a disclosing silicone [15]. Therefore, the surfaces need to be cleaned and conditioned sufficiently prior to adhesive cementation in order to obtain durable long-term bond strength [16,17,18,19,20,21].

Cleaning and conditioning of contaminated dental composite surfaces prior to adhesive cementation and the investigation of their influence to the bond strength was the objective of various previous studies [22,23,24,25,26,27,28,29,30,31,32,33]. Reports on cleaning methods of dental composites are limited to repair restorations [24,25,26,27,28,29,30,31,32,33,34]. There is a wide spectrum of cleaning methods for a bonding surface after contamination. Airborne particle abrasion or roughening with a burr lead to a poorer fit of the restorations resulting in poorer bond strengths [34,35,36,37].

To the best knowledge of the authors, cleaning methods of core build-up materials prior to adhesive cementation have not been examined to date. Due to the fact that the ingredients of these build-up materials differ from the composition of other composites used for direct restorations (e.g., fillings), the best cleaning method after contamination may also differ. The differences in the composites arise from their type (light curing and/or auto curing) and main focus of either mechanical feasibility or aesthetics. The selected build-up materials in this study were light and auto curing and focus mainly on mechanical feasibility because they are covered by other restorative materials.

Applicable cleaning methods might include using a pumice suspension with a rotating brush, intraoral airborne particle abrasion, using an air polishing powder or etching with phosphoric acid [38]. Scenarios for repairing composite fillings never investigated the influence of disclosing silicone remnants in combination with saliva contamination. Chemical bonding of core build-up materials might be achieved by either using a composite primer [39], which bonds to the organic phase, or by using a silane containing primer [22], which bonds directly to silicate-containing filler particles in the composite resin. Dentin primers might also act as a bond-mediating component to the core build-up material.

Therefore, the purpose of this study was to investigate the influence of different cleaning and surface conditioning methods on the tensile bond strength of luting resins to a core build-up material after contamination with saliva and a disclosing silicone. This study was designed to test the null hypothesis, that the described (1) cleaning methods and (2) conditioning methods have no influence on the bond strength of a luting resin to a contaminated core build-up material and its durability.

## 2. Materials and Methods

### 2.1. Specimen Preparation

Disc-shaped specimens (*n* = 384) of a core build-up material (Luxacore A3, DMG, Hamburg, Germany) were made. A 2-mL syringe was completely filled with the material of choice to create a composite cylinder with a diameter of 8.8 mm, which was sawed into 4 mm thick pieces after 10 min self-curing time. All specimens were wet polished with a rotating silicon carbide paper down to 600 grit and then stored for 3 weeks in 37 °C tap water to obtain water saturation and almost complete polymerization of the material.

### 2.2. Surface Contamination

Half (*n* = 192) of the specimens were contaminated by placing them with their bonding surfaces facing down into human saliva for one minute. The other half of the specimens remained uncontaminated. The donor of the saliva refrained from eating and drinking for 1.5 h prior to sampling. The saliva was used within 60 min after harvesting. The saliva was then removed from the specimens by spraying water for 15 s and then air drying for 15 s using an air blower with oil-free air. Afterwards, the bonding surfaces were pressed into a disclosing silicone (Fit Checker Black, GC Europe, Leuven, Belgium), removed after 5 min with any visible remnants manually detached.

### 2.3. Surface Cleaning

Four different cleaning methods were used to each treat 48 contaminated and 48 not contaminated specimens:

Phosphoric acid (37%, Etching Gel—Medium viscosity, DMG, Hamburg, Germany) was applied to the bonding surface. It was removed after 15 s by spraying water for 15 s and the bonding surface was dried with compressed, oil-free air for 15 s.

1. Pumice powder was mixed with a 0.9% NaCl-solution and was applied onto the bonding surface using a rotating brush for 15 s at a rotation speed of 2000 rpm. Afterwards, the pumice suspension was removed by spraying water for 15 s and then the bonding surface was dried with oil-free compressed air for 15 s.
2. Airborne particle abrasion: the bonding surface was marked with a red marker and then air-abraded with 50 µm alumina particles from a distance of 10 mm and a pressure of 0.5 bar until no colour remnants were visible. The remaining alumina particles were removed with spraying water for 15 s and then the bonding surface was dried with compressed, oil-free air for 15 s.
3. The dry bonding surface was cleaned with a sodium bicarbonate prophylaxis spray (Cavitron Prophy-Jet Prophy Powder, Dentsply DeTrey, Constance, Germany) using an air polisher (Cleanjet, Yoshida Dental, Tokyo, Japan) from a distance of 10 mm and a pressure of 2.5 bar for 15 s. The remaining prophylaxis powder particles were removed by spraying water for 15 s and then the bonding surface was dried with compressed, oil-free air for 15 s.

### 2.4. Surface Conditioning

The specimens in each of the four surface cleaning subgroups were randomly assigned to three smaller groups of 16 specimens per group. A different primer was used on each of the three smaller groups, as specified by the manufacturer:

1. A dentin adhesive (Optibond FL, Kerr Hawe, Bioggio, Switzerland), which is used as follows: The Optibond FL Primer was applied to the bonding surface using a disposable brush. After a dwell time of 30 s, the remaining liquid was removed by using an oil-free air blower for 15 s. Then, the Optibond FL Adhesive was applied using a disposable brush and was blown after 15 s using compressed, oil-free air for another 15 s. Afterwards, the adhesive was polymerized for 30 s with a dental curing light at a light intensity of 650 mW/cm^2^ (Demetron Optilux 501, Kerr, Danbury, CT, USA) from a distance of 10 mm.
2. A silane containing primer (Monobond Plus, Ivoclar Vivadent, Schaan, FL, Liechtenstein) was applied to the bonding surface using a disposable brush and after a dwell time of 60 s was dried with compressed, oil-free air for 15 s.
3. A composite resin primer (Ecusit-Composite Repair, DMG, Hamburg, Germany) was applied using a disposable brush. After a dwell time of 60 s is was gently blown using compressed, oil-free air for 15 s and light-cured for 20 s using a dental curing light at a light intensity of 650 mW/cm^2^ (Demetron Optilux 501, Kerr, Danbury, CT, USA) from a distance of 10 mm.

### 2.5. Bonding and Storage Conditions

Plexiglas tubes with an inner diameter of 3.2 mm (corresponds to a bonding surface of 0.08 cm^2^) were filled with dual-curing composite resin (Luxacore A3, DMG, Hamburg, Germany). After curing time (5 min), the filled tubes were bonded with a luting composite resin (Vitique White, DMG, Hamburg, Germany) to the core build-up composite surface using an alignment apparatus under a load of 750 g [40]. This apparatus ensured that the tube axis was perpendicular to the surface. After excess resin was removed, an air blocking gel (Vitique Try-In-Paste Transparent, DMG, Hamburg, Germany) was applied around the bonding margins. After 5 min, the bonded specimens were light-cured for 20 s from two opposite sides with a dental curing light at a light intensity of 650 mW/cm^2^ (Demetron Optilux 501, Kerr, Danbury, CT, USA), then further cured in a light-curing unit (Heraflash, Heraeus Kulzer, Hanau, Dresden, Germany) for 90 s, placed at room temperature for 10 min, and then stored in 37 °C tap water after removing the air blocking gel with water spray for 15 s. With regard to contamination presence, the four surface cleaning methods and three surface conditioning primers resulted in 24 test groups. For each test group, 16 specimens were bonded. Half of these bonded specimens were stored in tap water (37 °C) for 3 days and the other half for 150 days with artificial aging, where water storage was interrupted by 37,500 thermal cycles (5 to 55 °C) with a dwell time of 30 s. Composition and batch numbers of the materials are shown in Table 1.

To sum up the methods, a total of 24 test groups with 16 specimens per group were examined. The groups consisted of all possible unique contamination status, surface cleaning methods and conditioning primers. Test groups were divided in subgroups with 3 d short-term and 150 d long- term storage times with 8 specimens each. A visual overview of the different groups can be seen in Table 2.

### 2.6. Debonding and Statistical Analysis

At the end of the storage periods, tensile bond strength (TBS) was measured in a universal testing apparatus (Z010, Zwick, Ulm, Dresden, Germany) at a crosshead speed of 2 mm/min using a chain loop alignment which provided a moment-free axial load application. The fractured interfaces of the debonded specimens were examined using a light microscope (LM, Zeiss S7, Carl Zeiss AG, Oberkochen, Germany) at 30× magnification to calculate the fracture mode of each specimen as either adhesive or cohesive (failure in tube composite, the specimen composite or the bonding resin). Fractional allocation measured in percentages of adhesive and cohesive failure mode was possible in case of a mixed adhesive and cohesive failure mode. The arithmetic mean of both failure modes was then determined for each subgroup (*n* = 8). After sputtering a conductive gold layer with a thickness of approximately 15 nm as measured with a quartz crystal film thickness monitor (Leica EM QSG 100, Wetzlar, Germany), three representative samples of each group were examined in a scanning electron microscope (SEM, XL 30 CP, Philips, Kassel, Germany) with an acceleration voltage of 15 KeV.

Statistical analysis using the Shapiro-Wilk test showed that the data of some groups were not normally distributed. Therefore, further analysis was performed using the Kruskal-Wallis-Test followed by multiple pair-wise comparisons of the groups using the Wilcoxon rank sum test, corrected with the Bonferroni-Holm procedure for multiple comparisons within each rank sum test. The overall significance level was adjusted for multiple testing according to Bonferroni by the number of unique cleaning-conditioning combinations, resulting in the level of significance of *p* ≤ 0.0042.

## 3. Results

**Figure 1:**
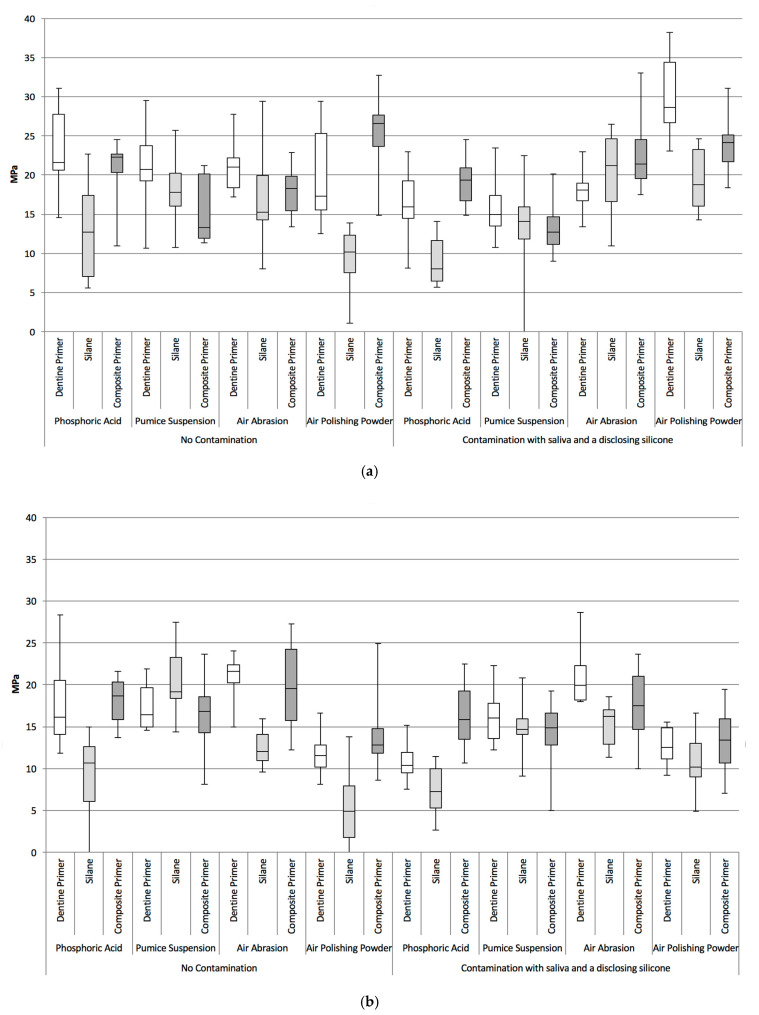
(**a**) Boxplots of TBS for all test groups after short-term storage (3 days); (**b**) Boxplots of TBS for all test groups after long-term storage in 37 °C tap water for 150 days with artificial aging.

Boxplots of TBS for all test groups after short-term storage are shown in Figure 1a and of all test groups after long-term storage in Figure 1b. The median TBSs of each test group are depicted in Table 2. The *p*-values of all performed group tests using the Wilcoxon rank sum test using Bonferroni-Holm correction can be found in Appendix A.

Generally, contamination resulted in lower median TBS, although it was statistically significant only in the test group treated with air polishing powder and silane conditioning after three days (Table A1). When comparing the storage conditions (short-term storage versus long-term storage), statistically significant (*p* ≤ 0.0042) lower TBS was detected only in the groups that had been cleaned with air polishing powder and treated with a dentin primer or a composite primer when in a not contaminated environment and any primer in a contaminated environment (Table A2).

The comparison of cleaning and conditioning methods in the long-term subgroup led to the following results: prior to conditioning the surface with a dentin adhesive, air-abrading the contaminated surface provided statistically significantly (*p* ≤ 0.0042) higher median TBS than air-polishing with prophylaxis powder, regardless of the contamination status. Moreover, cleaning the surface with phosphoric acid provided statistically significantly lower TBS than pumice suspension in a contaminated environment using a dentin primer. Phosphoric acid also led to a statistically significant lower TBS than air-abrasion in a contaminated environment using either dentin or silane primers. In fact, air abrasion exhibited the highest median TBS (16.2–19.9 MPa) in the contaminated subgroup regardless of the primer used (see Table 2). Using air abrasion, a statistically significant difference in median TBS in a contaminated environment was also observed in comparison to air polishing powder using a dentin primer. No statistically significant differences between the cleaning methods were observed after long-term storage when conditioning the bonding surface with a composite primer (Table A3).

Regarding conditioning methods in the long-term time period, a dentin primer resulted in statistically significantly lower median TBS than the other primers after cleaning with air-abrasion in an uncontaminated environment. Specimens treated with a combination of phosphoric acid and a composite primer achieved significantly higher bond strengths compared to the other test groups in a contaminated environment (Table A4).

**Figure 2:**
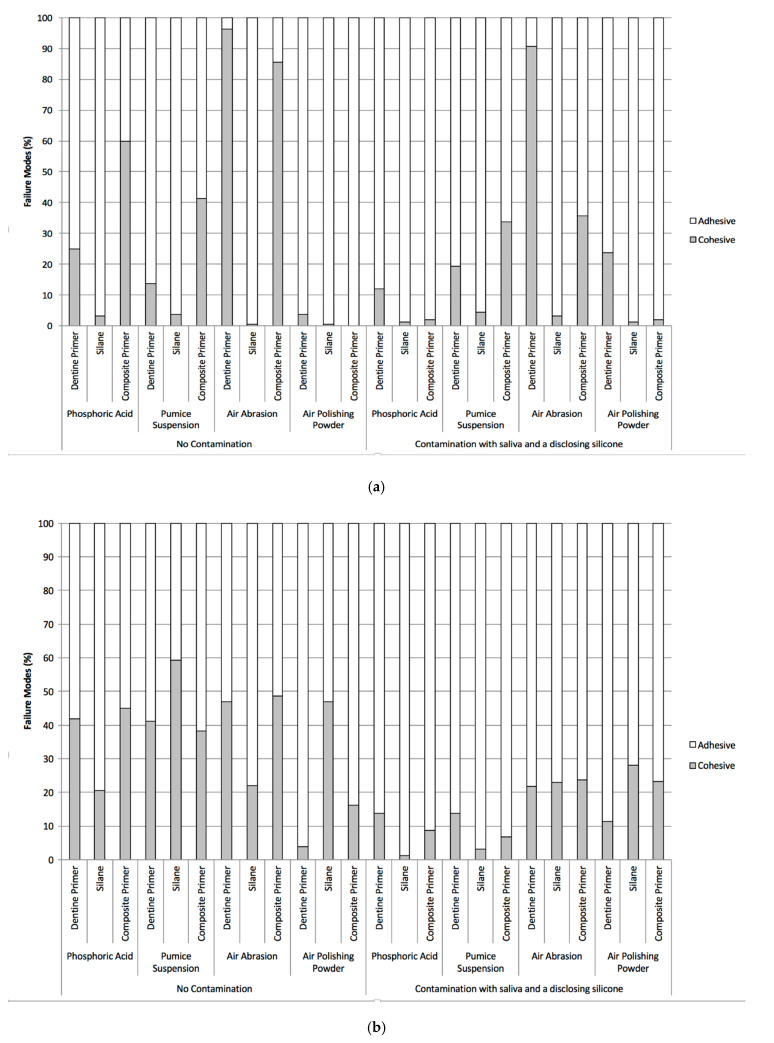
Type of bonding failure modes of test groups after (**a**) short-term storage in 37 °C tap water for 3 days and (**b**) for 150 days with artificial aging as identified with a light microscope at 30× magnification and calculated in percentage of the bonding area.

**Figure 3:**
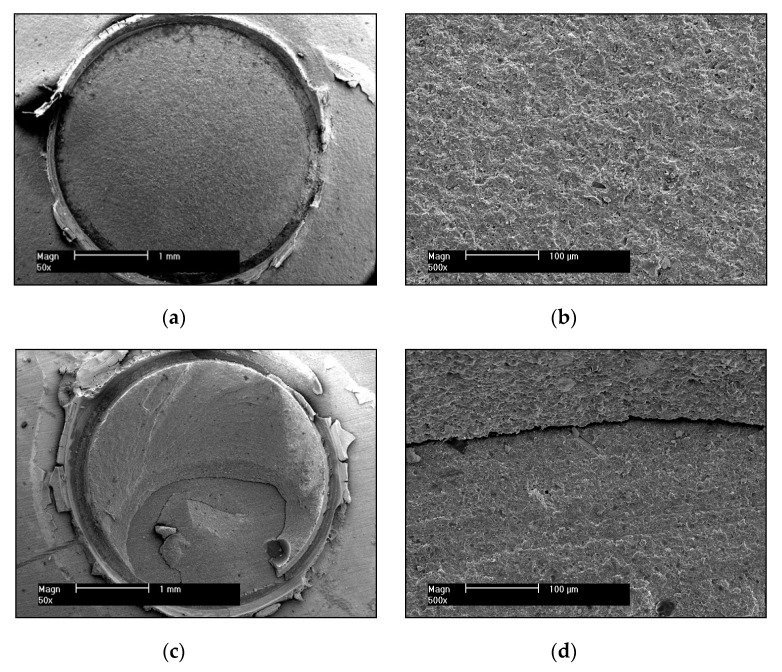
(**a**) A representative example of a purely adhesive failure mode in a debonded specimen. SEM micrograph: low magnification; (**b**) detailed SEM micrograph: high magnification of (**a**); (**c**) A representative example of a mixed adhesive and cohesive failure mode in a debonded specimen. SEM micrograph: low magnification; (**d**) detailed SEM micrograph: high magnification of (**c**).

The results of the failure mode analysis using light microscopy (LM) and scanning electron microscopy (SEM) are shown in Figure 2a for short-term storage and in Figure 2b for long-term storage and artificial aging. Contamination resulted in more adhesive bonding failures, whereas adequate cleaning resulted in more cohesive bonding failures. The examination of typical samples in the SEM verified the failure modes detected with the LM in all groups. Figure 3 shows SEM photographs with a typical example of a pure adhesive failure mode (Figure 3a,b) as well as a mixed adhesive and cohesive failure mode (Figure 3c,d). As can be seen in Figure 3a, the surface within the circle in the adhesive failure mode is flat and smooth, which is elaborated in the magnification seen in Figure 3b. Figure 3c, however, depicts a different scenario with a small smooth surface indicating an adhesive failure mode, which transitions into cohesive fracture lines running from the lower left area of the visible remnant of the luting resin to the right and therefore is most likely the origin of the failure in this specific sample. The transition zone between adhesive and cohesive failure modes can be seen in the high magnification SEM micrograph (Figure 3d). In this example, 40% of the failure is attributed to an adhesive and 60% to a cohesive mode.

## 4. Discussion

Four common cleaning methods were chosen for investigation in this study [12,15,41,42]. They were examined under two conditions—with contamination and without contamination—as well as two different time points—short-term (3 days) and long-term (150 days with artificial aging). Three surface conditioning methods were applied to each of the cleaning methods. Two of these three conditioning methods are principle methods for bonding to composite resins (bonding to the inorganic fillers or the organic phase) have previously been investigated in other studies for intra-oral repair procedures of composite fillings [23,39,43]. In most cases of adhesive cementation of dental restorations, conditioning the dentin with a dentin adhesive is necessary [44]. Hence, a dentin adhesive was chosen as a third option for conditioning build-up material before adhesive cementation, also because it is widespread and well known to almost all dentists. This study stands out as it investigated the influence of disclosing silicone remnants in combination with saliva contamination for various scenarios for repairing composite build-ups.

After long-term storage simulating the exposition in the oral environment, the bond strength decreased in 16 of the 24 test groups. This might be caused by water saturation and artificial aging, which leads to hydrolytic degradation of the used primers [45,46]. Considering the cleaning methods with contamination, air-abrasion and brushing with a pumice suspension lead to higher TBS than etching with phosphoric acid or using an air-polishing device in most scenarios. Air-abrasion causes pronounced micro roughening compared to air-polishing. Obviously, remnants of the used contaminations require a thorough mechanical treatment to be removed sufficiently.

Only one statistically significant difference in median TBS between contaminated and not contaminated in the 24 subgroups (air polishing powder cleaning and silane conditioning) could be observed. This is a limitation of our study, which is mainly caused by the low level of significance. Nevertheless, in our application scenario, i.e., bonding after the removal of the provisional restoration, cleaning is always necessary, since the adhesive surface would be contaminated by biofilm and/or remnants of the provisional cement.

Our findings compare and reproduce the results of other studies well, e.g., etching a ceramic bonding surface with phosphoric acid was not appropriate to remove remnants of a disclosing silicone and does not provide high TBS long-term. This can be explained by the inability of phosphoric acid to dissolve silicone oils [12], thus the combination of phosphoric acid and silane should be avoided. Another study showed similar results when cleaning a pre-etched lithium disilicate ceramic after contamination with a disclosing silicone [12]. The relatively low kinetic energy of the air-polishing device or the chemical composition might be responsible for an insufficient cleaning resulting in lower TBS long-term as our study showed the lowest median TBS of air polishing powder regardless of the contamination status and conditioning used in comparison to other cleaning methods.

The observed lack of potential of a silane to promote sufficient bonding of a luting resin to the core build-up material stands in contrast to the findings in other studies investigating intra-oral repairing procedures of composite fillings [22]. It might be explained by a different composition of core build-up materials compared to composites for direct fillings. For aesthetic and mechanical reasons, filling composites contain a relatively high amount of inorganic fillers made of silicates [43]. The composition of the used core build-up material contains a lower amount of silicates, which might result in fewer bindings of a silane to the bonding surface [47,48]. A similar effect was found in a recent study investigating resin bonding techniques to CAD/CAM resin composites [49].

To sum up, the following conclusions can be drawn from our study: (1) Cleaning a core build-up material bonding surface after contamination with saliva and a disclosing silicone with alumina particle air-abrasion or a rotating brush with pumice suspension resulted in statistically significantly higher TBS than cleaning with an air-polishing device or etching with phosphoric acid. (2) In some cases, using a silane containing primer for conditioning the bonding surface of a core build-up material resulted in statistically significantly lower TBS compared to conditioning with a dentin primer or a composite resin primer. Due to the observed statistically significant differences, the null hypothesis cleaning and conditioning methods have no influence on bond strength, must be rejected. After contamination with saliva and a disclosing silicone, bonding surfaces of a core build-up material should be cleaned with a rotating brush and a pumice suspension and conditioned with a dentin adhesive, which is conveniently required for adhesive bonding to tooth structure nonetheless. Using a less practical way, these surfaces could also be cleaned and conditioned with intraoral air-abrasion with alumina particles and a composite resin primer.

## Figures and Tables

**Table 1:** List of used materials and their characteristics.

Material	Main Composition	Manufacturer	Batch No.
Luxacore A3	Acrylate containing core build-up material	DMG	643862
Vitique White	Acrylate containing dual curing luting resin	DMG	632877 633912
Vitique Try-In-Paste	Glycerin based air blocking gel	DMG	635487
Fit Checker Black	Si/Sn cont. Silicone	GC	0409091
Etching Gel	37% Phosphoric acid/water cont. gel	DMG	637056
Ecusit Composite Repair	Acrylate containing composite primer	DMG	637728
Monobond Plus	Ethanol, water, silane methacrylate, phosphoric acid methacrylate, sulphide methacrylate	Ivoclar Vivadent	M35022
Optibond FL	Hydroxyethylmethacrylate, disodium hexafluorosilicate, ethyl alcohol	Kerr Hawe	25881E 25882E

**Table 2:** Median tensile bond strength (TBS) by cleaning, conditioning and contamination status.

Contamination Status.	Cleaning	Conditioning	Median TBS (MPa)	
			Short-Term (3 Days)	Long-Term (150 Days)
No Contamination	Phosphoric Acid	Dentin Primer	21.3	16.2
		Silane	12.7	10.7
		Composite Primer	22.2	18.7
	Pumice Suspension	Dentin Primer	20.8	16.5
		Silane	17.8	19.2
		Composite Primer	13.3	16.8
	Air Abrasion	Dentin Primer	21.0	21.6
		Silane	15.3	12.1
		Composite Primer	18.3	19.5
	Air Polishing Powder	Dentin Primer	17.3	11.6
		Silane	10.2	4.9
		Composite Primer	26.6	12.8
Contamination	Phosphoric Acid	Dentin Primer	15.9	10.3
		Silane	8.0	7.3
		Composite Primer	19.3	15.8
	Pumice Suspension	Dentin Primer	14.9	16.0
		Silane	14.1	14.6
		Composite Primer	12.7	14.9
	Air Abrasion	Dentin Primer	18.1	19.9
		Silane	21.2	16.2
		Composite Primer	21.4	17.5
	Air Polishing Powder	Dentin Primer	28.6	12.5
		Silane	18.8	10.2
		Composite Primer	24.1	13.4

## Appendix A

**Table A1:** Comparison of each test group with and without contamination using Wilcoxon rank sum test with Bonferroni-Holm procedure for multiple testing. Time periods were tested separately. Overall significance level adjusted for multiple testing according to Bonferroni (*p* ≤ 0.0042). Significant results are in bold and marked with (*).

	Short-Term (3 Days)			Long-Term (150 Days, Artificial Aging)		
	Dentin Primer	Silane	Composite Primer	Dentin Primer	Silane	Composite Primer
Phosphoric Acid	0.19487	0.38228	0.44180	0.00454	0.27863	0.44180
Pumice Suspension	0.13038	0.08298	0.50536	0.64538	0.03120	0.56324
Air Abrasion	0.10331	0.38228	0.04584	0.57374	0.03792	0.44180
Air Polishing Powder	0.01041	**0.00016 ***	0.27863	0.44180	0.05168	0.95913

**Table A2:** Comparison of unique contamination, cleaning and conditioning method combinations over time. Each unique combination was tested separately using Wilcoxon rank sum test with Bonferroni-Holm procedure for multiple testing. Overall significance level adjusted for multiple testing according to Bonferroni (*p* ≤ 0.0042). Significant results are in bold and marked with (*).

**No Contamination**	Phosphoric Acid	Dentin Primer	0.23450
		Silane	0.27863
		Composite Primer	0.06496
	Pumice Suspension	Dentin Primer	0.10490
		Silane	0.23450
		Composite Primer	0.87848
	Air Abrasion	Dentin Primer	0.87473
		Silane	0.08298
		Composite Primer	0.57374
	Air Polishing Powder	Dentin Primer	**0.00186 ***
		Silane	0.09241
		Composite Primer	**0.00186 ***
**Contamination**	Phosphoric Acid	Dentin Primer	0.01476
		Silane	0.32821
		Composite Primer	0.16053
	Pumice Suspension	Dentin Primer	0.79845
		Silane	0.56324
		Composite Primer	0.56324
	Air Abrasion	Dentin Primer	0.08298
		Silane	0.08298
		Composite Primer	0.08298
	Air Polishing Powder	Dentin Primer	**0.00016 ***
		Silane	**0.00274 ***
		Composite Primer	**0.00062 ***

**Table A3:** Comparison of cleaning methods by unique contamination status and conditioning combinations. Each unique combination and time period was tested separately using Wilcoxon rank sum test with Bonferroni-Holm procedure for multiple testing. Overall significance level adjusted for multiple testing according to Bonferroni (*p* ≤ 0.0042). Significant results are in bold and marked with (*).

			Short-Term (3 Days)			Long-Term (150 Days, Artificial Aging)		
			Phosphoric Acid	Pumice Suspension	Air Abrasion	Phosphoric Acid	Pumice Suspension	Air Abrasion
**No Contamination**	**Dentin Primer**	Pumice Suspension	0.79275	–	–	0.95913	–	–
		Air Abrasion	0.79275	0.79275	–	0.15646	0.03100	–
		Air Polishing Powder	0.79275	0.79275	0.79275	0.01399	**0.00559**	**0.00186 ***
	**Silane**	Pumice Suspension	0.26076	–	–	**0.00186 ***	–	–
		Air Abrasion	0.41795	0.64538	–	0.32821	**0.00186 ***	–
		Air Polishing Powder	0.53016	0.02098	0.02098	0.18604	**0.00186 ***	0.01504
	**Composite Primer**	Pumice Suspension	0.07111	–	–	0.60643	–	–
		Air Abrasion	0.15646	0.32821	–	0.72090	0.32106	–
		Air Polishing Powder	0.07483	0.02797	0.07111	0.14965	0.60643	0.14965
**Contamination**	**Dentin Primer**	Pumice Suspension	0.79845	–	–	**0.00218 ***	–	–
		Air Abrasion	0.53016	0.35175	–	**0.00047 ***	0.03375	–
		Air Polishing Powder	**0.00047 ***	**0.00062 ***	**0.00047 ***	0.10490	0.03375	**0.00047 ***
	**Silane**	Pumice Suspension	0.05986	–	–	**0.00823**	–	–
		Air Abrasion	**0.00326 ***	0.05688	–	**0.00186 ***	0.71299	–
		Air Polishing Powder	**0.00093 ***	0.04134	0.67420	0.22672	0.06041	0.06041
	**Composite Primer**	Pumice Suspension	0.04134	–	–	0.55752	–	–
		Air Abrasion	0.19263	0.01399	–	0.64538	0.53959	–
		Air Polishing Powder	0.04219	**0.00653 ***	0.50536	0.53959	0.64538	0.53959

**Table A4:** Comparison of conditioning approaches by unique contamination status and method combinations. Each unique combination and time period was tested separately using Wilcoxon rank sum test with Bonferroni-Holm procedure for multiple testing. Overall significance level adjusted for multiple testing according to Bonferroni (*p* ≤ 0.0042). Significant results are in bold and marked with (*).

		Short-Term (3 Days)			Long-Term (150 Days, Artificial Aging)		
		Dentin Primer—Silane	Dentin Primer—Composite Primer	Silane— Composite Primer	Dentin Primer—Silane	Dentin Primer—Composite Primer	Silane— Composite Primer
**No Contamination**	Phosphoric Acid	0.09744	0.95913	0.09744	0.01049	0.72090	0.00559
	Pumice Suspension	0.24079	0.24079	0.44180	0.29231	0.95913	0.27796
	Air Abrasion	0.19129	0.19129	0.57374	**0.00093 ***	0.57374	0.00443
	Air Polishing Powder	**0.00093 ***	0.16053	**0.00047 ***	0.02028	0.27863	0.02028
**Contamination**	Phosphoric Acid	0.00443	0.16053	**0.00047 ***	0.03792	0.00699	**0.00093 ***
	Pumice Suspension	0.57374	0.57374	0.57374	0.84485	0.84485	0.95806
	Air Abrasion	0.49231	0.06200	0.57374	0.00886	0.15734	0.44180
	Air Polishing Powder	**0.00186 ***	0.02214	0.04988	0.47710	0.79845	0.47710
